# Protein phase separation: new insights into cell division: Phase separation in cell division

**DOI:** 10.3724/abbs.2023093

**Published:** 2023-05-30

**Authors:** Hongdan Zheng, Wenyu Wen

**Affiliations:** Department of Neurosurgery Huashan Hospital the Shanghai Key Laboratory of Medical Epigenetics State Key Laboratory of Medical Neurobiology and MOE Frontiers Center for Brain Science National Center for Neurological Disorders Institutes of Biomedical Sciences School of Basic Medical Sciences Fudan University Shanghai 200032 China

**Keywords:** liquid-liquid phase separation, biomolecular condensate, cell division

## Abstract

As the foundation for the development of multicellular organisms and the self-renewal of single cells, cell division is a highly organized event which segregates cellular components into two daughter cells equally or unequally, thus producing daughters with identical or distinct fates. Liquid-liquid phase separation (LLPS), an emerging biophysical concept, provides a new perspective for us to understand the mechanisms of a wide range of cellular events, including the organization of membrane-less organelles. Recent studies have shown that several key organelles in the cell division process are assembled into membrane-free structures via LLPS of specific proteins. Here, we summarize the regulatory functions of protein phase separation in centrosome maturation, spindle assembly and polarity establishment during cell division.

## Introduction

Recently, liquid-liquid phase separation (LLPS), a physical-chemical paradigm, has gradually become prosperous in the field of life science [ 1– 4] . Basic cellular activities are mainly carried out in distinct cellular compartments with unique biological functions, some of which are not enclosed with membranes and might be formed tempo-spatially. Such membrane-free organelles, including the nucleolus [5], ribonucleoprotein granules [ 6, 7] , and signaling machinery beneath the plasma membrane [8], have been found to assemble via LLPS of their constituent proteins and/or nucleic acids into biomolecular condensates with liquid-like behavior. The driving force for organizing biomolecular condensates involves multivalent interactions between constituent proteins/nucleic acids and self-association via oligomerization domains or intrinsically disordered regions [ 4, 9, 10] . The distinct physical-chemical properties of these biomolecules enable them to autonomously coacervate from the surrounding cytosol or nucleosol in a similar manner that oil demixes from water. Increasing studies have demonstrated the essential roles of biomolecular condensates in regulating various cellular events, including DNA transcription and translation [ 11, 12] , signal transduction [13], asymmetric cell division [14], as well as reproductive development [ 15, 16] . Disturbance of physiological LLPS process or pathological phase separation/transition is closely related to tumor development [ 17– 19] and neurodegenerative diseases [ 20, 21] . Overall, phase separation provides a new perspective for investigating and understanding the mechanisms of distinct physiological and pathological phenomena.

As the foundation for individual growth and development, as well as tissue repair and regeneration after damage, the process of cell division involves a wide range of subcellular organelles with specific components, such as centrosomes, chromosomes, spindles, and polarized protein complexes beneath the plasma membrane. Centrosomes are usually located near the central region of cells that act as the major microtubule organization centers (MTOCs) in most eukaryotic cells [ 22– 24] . As the main mechanical support during cell division, spindle microtubules nucleate at centrosomes to drive chromosome arrangement in the metaphase plate. After cell division, a mother cell evenly distributes genetic materials into two daughter cells and this process relies on precise control of spindle organization and localization. Whereas asymmetric cell division may also occur for stem cells and progenitor cells, during which cell fate determinants and polarized protein complexes are transiently located on the basal and apical cortex of cells, respectively, and then unevenly segregated into two daughter cells with distinct fates [25]. Intriguingly, all the above mentioned subcellular organelles are tempo-spatially organized membrane-less compartments during cell division. Increasing studies have revealed a regulatory role of LLPS in organizing these key structures via the formation of specific biomolecular condensates during cell division, and some of them have been summarized in excellent reviews [ 26– 28] . The goal of this article is to give a brief overview of newly discovered biomolecular condensates involved in cell division, emphasizing how LLPS of biomolecules regulates centrosome maturation, spindle assembly as well as cell polarization.

## Phase Separation in Centrosome Biogenesis and Maturation

**Figure 1:**
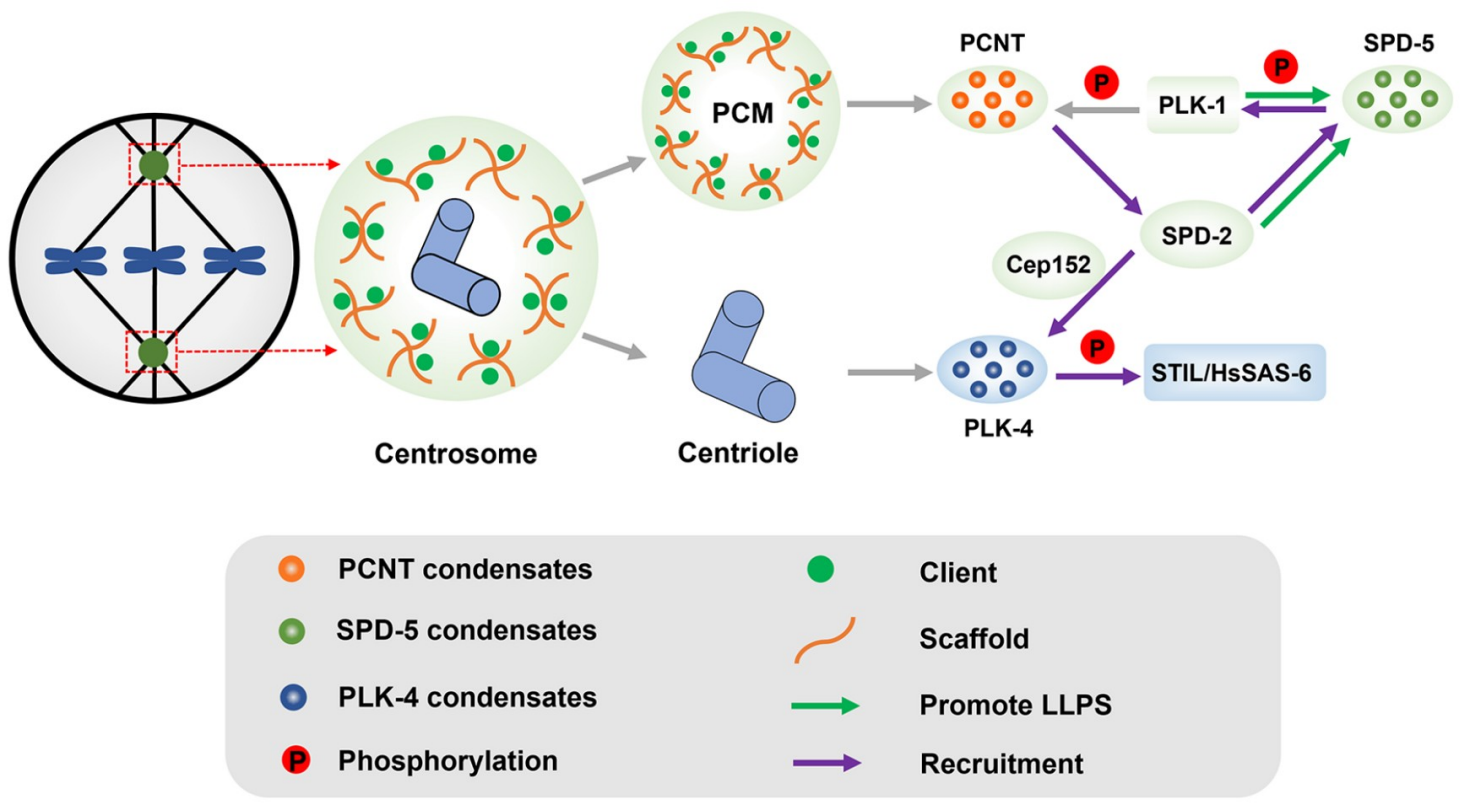
Figure 1 Phase separation in centrosome biogenesis and maturation The centrosome is composed of centrioles and PCM. The key constituent proteins of PCM include PCNT (human), Cep192 (human)/SPD-2 ( C. elegans), as well as SPD-5 ( C. elegans), and PCNT and SPD-5 have been found to undergo LLPS to form biomolecular condensates. PCNT and SPD-5 can be phosphorylated by PLK-1, and the phosphorylation of SPD-5 facilitates its phase separation. SPD-2 acts as a scaffold to recruit the protein kinase PLK-4, and then PLK-4 undergoes LLPS to facilitate recruitment and phosphorylation of the downstream effector STIL/HsSAS-6 complex to promote centriole replication.

Centrosomes play essential roles in spindle assembly and cell polarity initiation [ 22, 29] , and malfunction of centrosomes results in tumorigenesis and developmental disorders [ 30, 31] . The core structure of the centrosome consists of a pair of mutually perpendicular centrioles and the pericentriolar material (PCM) ( Figure 1). Centrioles ensure the stability of centrosome replication, whereas the microtubule organization activity of the centrosome is primarily determined by PCM, which serves as the scaffold to recruit distinct nucleators and regulators involved in microtubule nucleation and spindle assembly [32]. Intriguingly, centrioles only organize a small size of PCM during interphase; however, there is a dramatic and rapid expansion of PCM when the cells enter mitosis [33]. In the past decades, great progress has been made in deciphering the main constituent proteins of PCM, including pericentrin (PCNT) in human [34], SPD-2 (also known as Cep192 in humans) and SPD-5 in *C*. *elegans* [ 35, 36] . During centriole-to-centrosome conversion, the recruitment of these PCM components around centrioles is particularly critical because they act as scaffolds for organizing other proteins required for PCM expansion and centrosome biogenesis [ 37, 38] . Kinases, such as Polo-like kinases (PLKs), were also found to regulate the duplication and segregation of centrosomes during cell division [ 39, 40] . Though the mechanisms of centrosome biogenesis and maturation have been illustrated at the molecular level, it is still not clear how to prevent the crowded constituent proteins from dispersing without the enclosing membrane. Interestingly, some PCM components and regulators have been found to undergo LLPS, thus promoting centrosome maturation, microtubule nucleation as well as spindle assembly.

The evolutionarily conserved scaffold protein PCNT is preferentially recruited to centrosomes during mitosis, where it plays a critical role in PCM organization and centrosome maturation by recruiting other PCM core components [ 37, 41] . The protein level of PCNT needs to be tightly regulated. Loss-of-function mutagenesis of PCNT leads to severe congenital disorders including primordial dwarfism [42] and Seckel syndrome [43], possibly due to impaired asymmetric divisions in embryos and adult stem cells, resulting in decreased body size [44]. Whereas elevated PCNT expression level disrupts ciliary protein transport to centrosomes and signal transduction, eventually resulting in Down syndrome [11]. As a dynein cargo, cytoplasmic PCNT can be transported to centrosome along microtubules and contribute to microtubule nucleation through recruiting the conserved nucleation factor gamma-tubulin ring complex (γ-TuRC) [ 45, 46] . As a result, the majority of endogenously expressed PCNT is recruited to the surrounding of centrioles. Structural studies revealed that PCNT and PCNT-like proteins form elongated fibrous structures, which extend radially from the centrioles to support the organization of PCM and centrosome [ 41, 47] . It has been recently demonstrated that PCNT forms droplet-like granules around centrioles during the late G2/early M phases of cell division [48]. The N-terminal of PCNT, enriched in evolutionarily conserved coiled-coils and low complexity regions, drives condensate formation in a concentration-dependent manner. These liquid-like granules exhibit typical properties of biomolecular condensates and are sensitive to the treatment with 1,6-hexanediol, suggesting that PCNT condensates are maintained by weak hydrophobic interactions [48]. PCNT condensates move towards centrosomes in a dynein- and microtubule-dependent manner. Interestingly, PCNT condensates gradually transition from liquid to gel-like state at centrosomes and exhibit centrosome-like activities, including selective recruitment of other PCM components (e.g., γ-tubulin and Cep192/SPD-2) to promote microtubule nucleation and PCM scaffold organization. It was noted that the centrosomal gel-like PCNT is insensitive to the treatment with 1,6-hexanediol, implying that the liquid-to-gel transition of PCNT condensates at mitotic centrosomes may change its biophysical properties [48]. However, the consequence of disrupting PCNT phase separation has not yet been investigated, and whether it influences PCM organization and microtubule nucleation remains unknown.

An intriguing question is how the membrane-less centrosomes recruit and organize tubulin for microtubule nucleation and assembly? A potential candidate is the PCM scaffold protein CDK5RAP2 (Cnn in *Drosophila*), and its functional homolog in *C*. *elegans* is SPD-5 [49]. During centrosome maturation, centriole-localized Cep192/SPD-2 further recruits SPD-5 into centriole. After being recruited, SPD-2 and SPD-5 mutually depend on each other for their stable localization at PCM, then SPD-5 and Cep192/SPD-2 gradually accumulate at the surrounding of centrioles and act as scaffolds for the recruitment of other constituent proteins to organize PCM [ 35, 50] . The centrosome-localized SPD-5 further recruits numerous downstream effectors such as the Polo-like kinase PLK-1 [51], the microtubule-stabilizing protein TPX2 [52], and the microtubule polymerase ZYG-9 [53]. A recent study indicated that SPD-5 targets centrioles through the centriole localization (CL) domain, and PLK-1-mediated phosphorylation of SPD-5 enhances the interaction between CL and the centriolar protein PCMD-1, promoting the organization and expansion of PCM scaffold [54]. On the other hand, PLK-1 could phosphorylate PCNT to initiate centrosome maturation by recruiting other constituent proteins to form PCM lattice, and then PCM lattice gradually becomes enlarged and ultimately functions as the mitotic spindle pole for microtubule nucleation [55]. Interestingly, another study suggested that SPD-5 could coordinate with γ-TuRC to promote PCM assembly and microtubule nucleation in the absence of centrioles. This event is independent of the mitotic kinase regulation, suggesting the intrinsic capability of SPD-5 in regulating MTOC function [56]. It was recently found that SPD-5 undergoes LLPS *in vitro*, forming amorphous droplets with dynamic properties, and these droplets gradually transition from liquid to gel-like state over time [57]. *In vitro* experiments showed that the presence of PLK-1 and SPD-2 could enhance the formation of SPD-5 condensates, whereas the recruitment of ZYG-9 and TPX2 into SPD-5 condensates is required for generating microtubule asters, as these biomolecular co-condensates enrich tubulin, microtubule-associated proteins as well as regulatory proteins [57]. Overall, these *in vitro* works support a hypothesis that these centrosome components might regulate centrosome biogenesis and maturation via formation of biomolecular condensates. However, rigorous *in vivo* studies are needed to define the biophysical properties of these biomolecular condensates and clarify the correlation between LLPS properties and physiological functions of these centrosome components.

PLK family is a key regulator of cell division, and dysregulation of PLK family members is associated with abnormal cell division and tumorigenesis [ 39, 58] . Recent studies have demonstrated that PLK kinases including PLK-1 and PLK-4 are involved in the replication and segregation of centrosomes, as well as the assembly of bipolar spindle [ 59– 62] . During centriole replication, PLK-4 is a primary regulator of initiating centriole assembly and organization, in cooperation with other proteins including Cdk2, CP110 and HsSAS-6 [ 62, 63] . Interestingly, Cep192/SPD-2 and Cep152 (Asterless in *Drosophila*) act sequentially as scaffolds for the recruitment of PLK-4 into the mother centriole, and their homologous N-terminal acidic-α-helix and N/Q-rich motifs competitively interact with the cryptic polo-box (CPB) of PLK-4. Disruption of the interaction between either Cep192 or Cep152 with PLK-4 is sufficient to weaken PLK-4-dependent centriole duplication and leads to cell-proliferation defect [64]. This competitive interaction ensures that Cep192 and Cep152 hierarchically mediate the recruitment of PLK-4 into centrioles and Cep192 combined with PLK-4 earlier than Cep152 in this process. After being recruited, Cep152 could be phosphorylated by PLK-4 and act together with PLK-4 to initiate centriole replication. Meanwhile, PLK-4 phosphorylates the downstream effector STIL to facilitate the recruitment of HsSAS-6, a major component of the centriole [ 64, 65] . The coordination of PLK-4, STIL, and HsSAS-6 initiates offspring centriole assembly and parental centriole replication [ 66, 67] . Recently, it was found that PLK-4 undergoes phase separation upon autophosphorylation of its CPB domain, generating nanoscale-spherical condensates which could efficiently capture and enrich binding partners from the surrounding environment [68]. Moreover, self-phosphorylation of PLK-4 accelerates its dissociation with Cep152 for binding to and phosphorylating STIL, leading to the recruitment of HsSAS-6 to form the STIL/HsSAS-6 complex [68]. As the concentration of the PLK-4/STIL/HsSAS-6 complex needs to reach a certain threshold to initiate centriole duplication, the formation of PLK-4 condensates upon autophosphorylation is essential to recruit a sufficient amount of STIL/HsSAS-6 complex to drive centriole biogenesis and replication [ 68– 70] . In recent years, emerging studies have found that PLK-4-targeted inhibitors destroy the activity of centriole in cancer cells and inhibit the proliferation of cancer cells, and PLK-4 is regarded as a potential anti-cancer target [ 71, 72] . Is the anti-cancer potential of PLK-4 inhibitor related to the phase separation properties of PLK-4? In other words, could LLPS provide a new strategy for cancer treatment? More studies are required to answer these questions.

## Phase Separation in Spindle Assembly and Orientation

**Figure 2:**
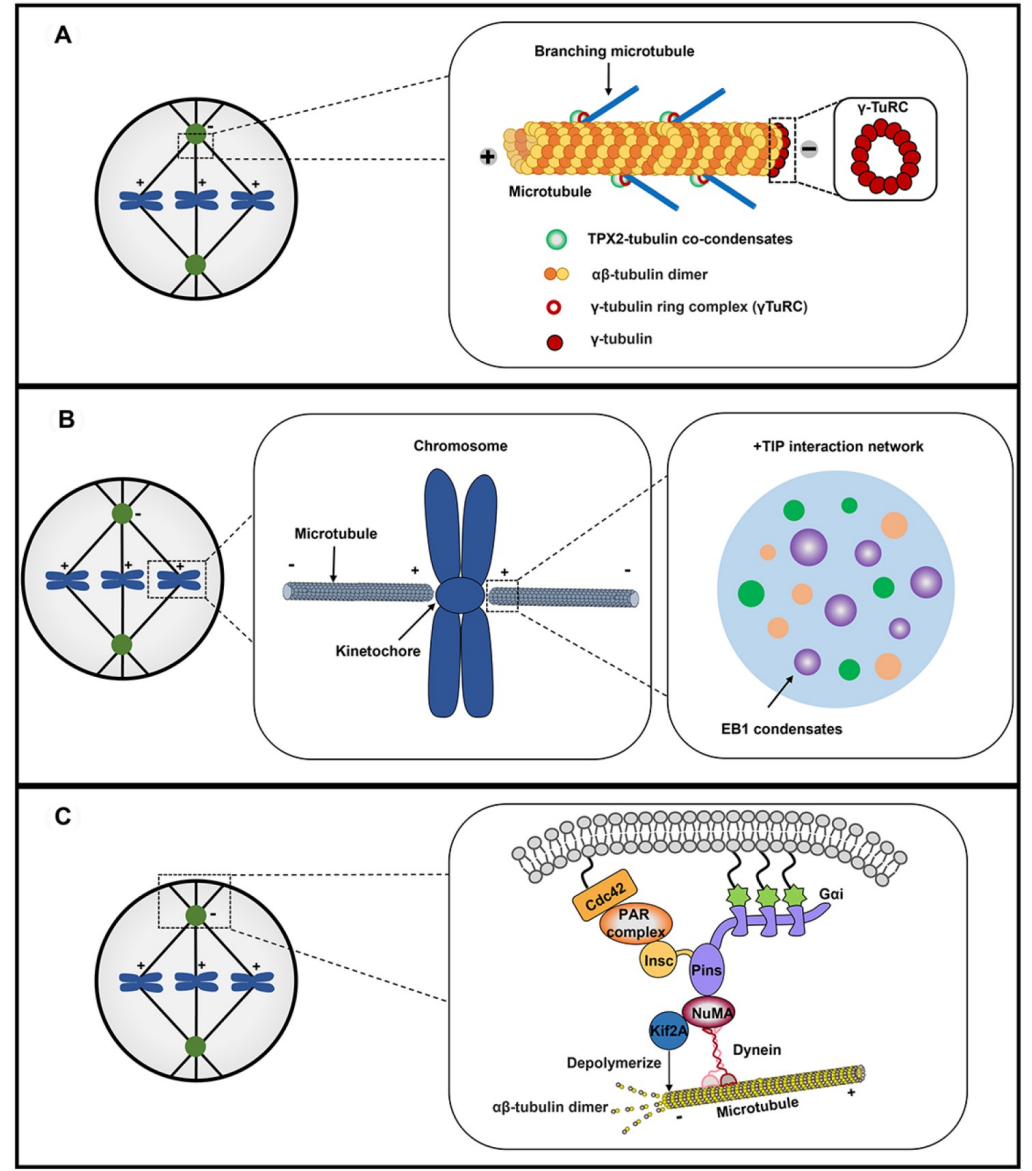
Figure 2 Phase separation in spindle assembly and orientation (A) TPX2 undergoes phase separation and preferentially forms condensates with αβ-tubulin dimers in pre-existing microtubules. Branching microtubules nucleate along pre-existing microtubules to establish microtubule networks, and TPX2-tubulin condensates provide the basis for efficient branching microtubule nucleation. (B) EB1 undergoes phase separation via multivalent weak interaction-induced self-association, and EB1 condensates recruit more +TIP partners for efficient microtubule plus-end tracking to regulate mitotic microtubule dynamics and accurate chromosome segregation. (C) NuMA undergoes phase separation to spontaneously form droplet-like granules, and NuMA condensates are transported into spindle poles along microtubules. In mitotic spindle poles, NuMA condensates recruit the microtubule depolymerization protein Kif2A for the depolymerization of spindle microtubules to regulate microtubule dynamics and spindle organization.

The spindle is a membrane-less, highly self-organized and dynamic organelle existing during the prophase to telophase of cell division, which is composed of microtubules, molecular motors, and a series of supramolecular machineries [23]. The precise assembly and orientation of spindle are essential for regulating chromosome segregation and cell fates [ 23, 73, 74] . Specifically, the duplicated chromosomes line up at the metaphase plate where they are tightly attached to the spindle microtubules through kinetochores. During the anaphase, the spindle pulls the sister chromatids toward the opposite poles, with the genetic material equally segregated. In addition, astral microtubules could also transmit cortical polarity signal to the centrosome to mediate asymmetric division [75]. Recent studies have revealed that some key regulators of mitotic spindle exhibit phase separation properties, and we highlight the essential roles of TPX2, EB1 as well as NuMA in regulating organization and localization of microtubule spindles in Figure 2.

Microtubules from various sources are nucleated by γ-TuRC to coordinate spindle assembly, which also localizes at the surface of pre-existing microtubules to promote branching microtubule nucleation with the help of other nucleators [ 75– 77] . Xklp2 (TPX2), which contains the γ-TuRC nucleation activator motif, is regarded as a key factor in γ-TuRC-dependent microtubule nucleation, and thus regulates spindle integrity, genomic stability as well as tumor development [ 77, 78] . In *Xenopus* egg extracts, TPX2 could facilitate branching microtubule nucleation once released from importin by Ran-GTP [79]. In *C*. *elegans* embryos, the spindle length decreases with each mitotic division, and TPXL-1 (the homolog of TPX2) is involved in setting spindle length of the first mitosis [80]. Recently, King *et al*. [81] noticed that the structural characteristic of TPX2 is similar to phase-separated proteins, and TPX2 forms spherical condensates together with αβ-tubulin dimers *in vitro* and in the isolated *Xenopus* egg cytosol. TPX2/tubulin condensates preferentially emerge in pre-existing microtubules, and subsequently, the branching microtubules nucleate along the pre-existing microtubules to establish branching microtubule networks [8]. Phase separation of TPX2 and tubulin guarantees 10- *fold* improvement of branching microtubule nucleation efficiency, therefore TPX2-tubulin co-condensation might underlie the all-or-nothing activation of branching microtubule nucleation [81]. Importins-α/β heterodimer inhibits TPX2-tubulin co-condensation *in vitro* and reduces TPX2-mediated microtubule nucleation in the isolated *Xenopus* egg cytosol in a concentration-dependent manner [81]. However, it has not been explored whether importins-α/β heterodimer inhibits TPX2-tubulin co-condensation and branching microtubule nucleation in physiological condition. Generally speaking, phase separation of microtubule nucleation effectors provides a structural basis for improving microtubule nucleation efficiency, and it might be an essential regulator for spatially coordinated spindle assembly during cell division.

EB1 is a microtubule plus-end tracking protein (+TIP), which has the intrinsic capability to bind with growing microtubule plus-ends and recruit +TIP partners to form an interaction network [82]. Meanwhile, EB1 is an evolutionarily conserved microtubule-plus-end-tracking protein; its N-terminal region includes the calponin homology domain for microtubule plus-end binding, and its C-terminal region includes the coiled-coil domain for EB1 dimerization and +TIPs interaction [ 83, 84] . However, the physical-chemical properties of the EB1 network and the molecular mechanisms of how EB1 and other +TIPs track microtubule plus-end remain unclear. A latest study revealed that EB1 undergoes phase separation via multivalent weak interaction-induced self-association in living cells and *in vitro*, which contributes to recruiting +TIP partners for efficient microtubule plus-end tracking. In addition, EB1 condensates could concentrate αβ-tubulin dimers *in vitro* conditions, indicating they act as dynamic storehouse for αβ-tubulin dimers to regulate spindle microtubules elongation and spindle organization [85]. Interestingly, LLPS-deficient EB1 mutants do not interfere the interaction between EB1 and other +TIP proteins, but result in mitotic chromosome separation defect and spindle localization error, suggesting that EB1 phase separation might be critical for mitotic microtubule dynamics and accurate chromosome segregation [85]. Taken together, these findings indicated that LLPS-driven EB1 condensates provide a novel molecular mechanism for tempo-spatially accurate regulation of microtubule plus-end dynamics during cell division, to maintain the quality control of cell renewal and genome stability. What’s more, it has been shown that the post-translational modification of EB1 is critical for chromosome stability maintenance [86]. The molecular mechanisms of phase separation-dependent and phase separation-independent EB1 protein in regulating microtubule dynamics and genome stability can be explored in depth, which may help provide novel therapeutic strategies for chromosome instability-induced diseases.

NuMA (nuclear mitotic apparatus; Mud in *Drosophila*) is an evolutionarily conserved multifunctional protein with cell cycle-dependent distribution, which plays critical roles in nuclear formation, mitotic spindle assembly and orientation [ 87, 88] . During interphase, NuMA predominantly localizes in the nucleus due to its C-terminal nuclear localization signal, whereas it transfers to spindle poles and cell cortex when entering mitosis. By forming a complex with dynein-dynactin motor, NuMA acts as a linker anchoring spindle microtubules into spindle poles [89]. A study in human oocytes showed that NuMA is enriched at microtubule negative ends, where it recruits cytoplasmic dynein and cross-links microtubules for spindle pole organization in the absence of MTOCs [90]. Depletion of NuMA or inhibition of dynein stops extension of the microtubule negative ends and the spindle poles become fully defocused. In addition, the dynein-dynactin-NuMA clusters exhibit dynamic asymmetric cortical position and generate cortical pulling forces to determine the spindle orientation and localization [ 91, 92] . PLK-1 negatively regulates the position of cortical dynein by controlling the interaction between dynein-dynactin and NuMA [93]. A recent study suggested that NuMA regulates the mitotic spindle assembly and function via its C-terminal tail-mediated phase separation [94]. During mitosis, NuMA spontaneously forms droplet-like granules, which are transported into spindle poles along microtubules. Spindle pole-localized NuMA condensates do not directly participate in acentrosomal microtubule nucleation, but are involved in concentrating tubulins and sorting acentrosomal microtubules into spindle microtubule arrays to facilitate mitotic spindle assembly [94]. NuMA condensates also recruit Kif2A, a microtubule depolymerization protein, to spindle poles for the depolymerization of spindle microtubules, thereby regulating the microtubule dynamics and spindle length [94]. On the other hand, NuMA condensates are negatively regulated by cell cycle kinase Aurora A-mediated phosphorylation. Phosphorylated NuMA possesses an enhanced mobility enabling it to shuttle between mitotic spindle poles and cellular cortex, thus contributing to mitotic spindle orientation [94].

## Phase Separation in Asymmetric Cell Division

**Figure 3:**
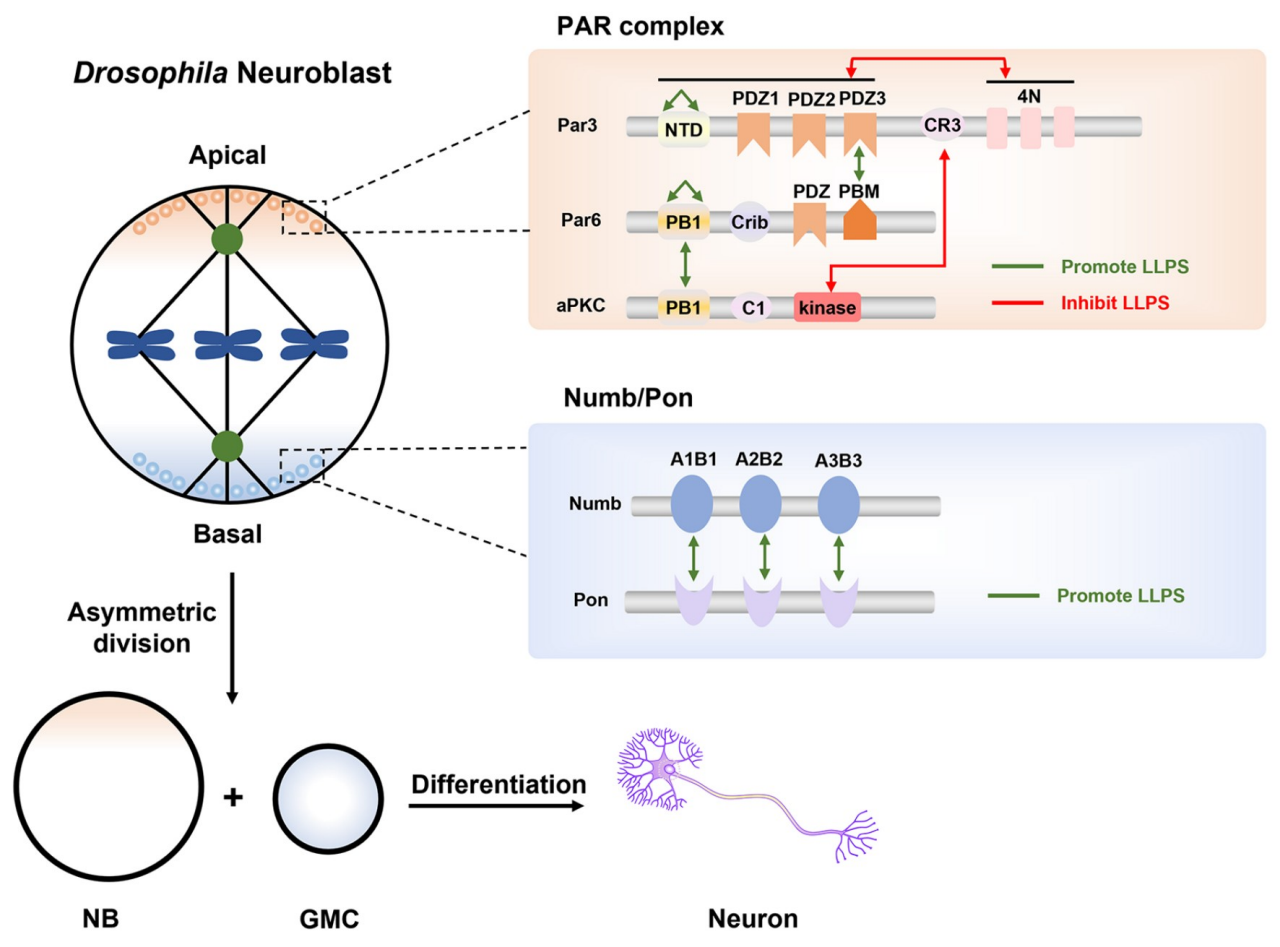
Figure 3 Via ACD, a *Drosophila* NB produces two daughter cells including one NB and one GMC with distinct fates, the latter differentiating into neurons Different protein complexes are concentrated at limited membrane regions by multivalent interaction-mediated LLPS. PAR complex and related proteins condense on the apical cortex in the form of liquid-like granules, while cell fate determinants including Numb and its adaptor Pon are highly concentrated on the basal cortex of cells. Interactions between different domains can facilitate or inhibit protein phase separation. Self-association and specific intermolecular interactions (indicated by green arrows) promote LLPS, and posttranslational modifications ( e.g., phosphorylation) and intramolecular autoinhibition (indicated by red arrows) inhibit LLPS of protein complexes. NTD (N-terminal domain); PDZ (PSD-95/DLG/ZO-1); CR (conserved region); PB1 (Phox and Bem1); Crib (CDC42/Rac-interactive binding); PBM (PDZ-binding motif); PTB (phosphotyrosine binding).

In some cases, a cell generates two distinct daughter cells after division, a process called asymmetric cell division (ACD) [95], and dysfunction of ACD is associated with developmental abnormalities and tumorigenesis [ 96, 97] . ACD is an essential mechanism to generate diversity during development of multicellular organisms, which mainly consists of four steps: symmetry breaking, polarity establishment, spindle orientation, and protein segregation [25]. Polarity establishment is a critical step of ACD, which usually refers to the asymmetry of intracellular protein distribution [98]. Taking the model system *Drosophila* Neuroblast (NB) as an example, during its asymmetric division, one NB produces two daughter cells with distinct fates, one self-renewed NB and one ganglion mother cell (GMC) which then differentiates into neurons ( Figure 3). After the symmetry of cell is broken, cell fate determinants and polarity protein complexes gradually move towards the opposite cell cortex to establish apical-basal polarity [ 25, 97, 99] . Specifically, cell fate determinants Numb, Prospero (and its mRNA), Brat, mRNA binding protein Staufen, and their adaptor proteins adaptor of Numb (Pon) and Miranda distribute on the basal membrane, whereas the PAR (Par3/Par6/aPKC) polarity complex, Inscuteable (Insc), and Pins are apically localized ( Figure 3) [ 100– 108] . Then the microtubule anchored protein Mud ( *Drosophila* homologue of NuMA) bridges the mitotic spindle with apical cortex by forming a complex with the apical PAR/Insc/Pins/Gαi complex and the Dynein/Dynactin motor [ 107, 109– 111] . The pulling force generated via minus-end-directed Dynein/Dynactin on the astral microtubules orients the spindle along the polarity axis [ 110, 112– 115] , thus leading to uneven segregation of apical and basal proteins (including cell fate determinants), eventually giving rise to two cells with distinct fates [116]. However, how these fate determinants and polarized proteins are localized on specific cortex regions rather than distributed throughout the cell is poorly understood. Recent studies have demonstrated that phase separation is involved in regulating the distribution of these protein complexes, which provides a new angle to understand the regulatory mechanism of cell polarity establishment.

In the interphase, the Notch inhibitor Numb is evenly distributed on the cell cortex and cytoplasm of NBs. When entering mitosis, Numb and its adaptor Pon form a concentrated crescent on the basal cortex of cells, and then segregate into the basal daughter GMC to drive its differentiation [ 108, 117] . Recent findings indicated that LLPS of proteins may be an underlying mechanism to regulate the uneven localization of cell fate determinants [118]. During *Drosophila* NB asymmetric division, the PTB domain of Numb specifically recognizes repeat motifs in the N-terminal of Pon in an atypical pattern, and such multivalent interaction induces the Numb/Pon complex to undergo phase separation [118]. Numb/Pon complex spontaneously assemble membrane-free dense liquid-like structures both *in vitro* and in living cells. Proteins within the dense liquid phase are not in a captured state, but freely and dynamically exchanging with the proteins within or outside the condensates [118]. Importantly, Numb/Pon condensates could be dispersed by competing peptides of Numb PTB domain and 1,6-hexanediol. Disruption of Numb/Pon condensates interferes with Numb basal localization and inhibits the Notch signaling pathway during ACD of *Drosophila* NBs, ultimately resulting in tumor-like NB hyperproliferation phenotype [118]. For the first time, this study revealed that phase separation could be one potential mechanism for polarized localization of biomolecules, which regulates polarity-related cellular events including asymmetric cell division.

The evolutionarily conserved PAR complex contains Par3 (Bazooka in *Drosophila*), Par6, and atypical protein kinase C (aPKC). At the beginning of ACD of *Drosophila* NBs, the PAR components are uniformly distributed on the membrane or in the cytoplasm, and then PAR proteins gradually emerge on the apical cortex of cells, forming a crescent at metaphase. Meanwhile, the cell fate determinants and their adaptor proteins are enriched at the basal cortex of the cell upon aPKC-mediated phosphorylation, thereby establishing the apical-basal cell polarity [ 95, 119, 120] . A recent study found that PAR proteins condense on the apical cortex in the form of liquid-like granules [14]. The NTD domain-mediated oligomerization of Par3 is the driving force for its phase separation both *in vitro* and in physiological environments. Par6 could be recruited and enriched into Par3 condensates as its C-terminal motif specifically binds to the PDZ3 domain of Par3, which further promotes the phase separation capability of Par3 [14]. Kinase aPKC could also be recruited into Par3/Par6 condensates as an inactive client, and then be transported to apical cortex at mitosis. The apically localized aPKC would be activated with unknown mechanism, leading to phosphorylation of cell fate determinants and their adaptors to preclude their apical localization. However, aPKC-mediated phosphorylation of Par3 results in dispersion of Par3/Par6 condensates, and the PAR proteins uniformly distribute on the cortex or cytoplasm of the apical daughter after cytokinesis, ready for another cycle of ACD. Interfering with the formation of Par3/Par6 condensates disrupts the establishment of apical-basal cell polarity during ACD of *Drosophila* NBs, eventually resulting in neuronal lineage developmental defects [14]. It is suggested that protein phase separation mediated by multivalent interactions may be a universal principle regulating the establishment of cell polarity, and protein LLPS provides a rational explanation for how polarized proteins achieve selective condensation while remain highly dynamic.

## Conclusion and Perspective

Liquid-liquid phase separation is considered as a fundamental mechanism for the assembly of membrane-less subcellular organelles, which are liquid-like, highly dynamic and inhomogenous structures containing unique proteins and/or RNAs components that spontaneously demix from the surroundings. Within such spatiotemporally organized individual compartments, specific biomolecular interactions and reactions are executed for precise regulation of various intracellular events. Studies related to the biogenesis and maturation of centrosome, the assembly and orientation of spindle, and the establishment of cell polarity have suggested that LLPS is a common strategy to achieve dynamic condensation of specific biomolecules to regulate cell division process in multicellular organisms. In addition, increasing evidence has suggested a critical role of LLPS in bacterial cell division [ 121, 122] . During the asymmetric cell division of the model bacterium *Caulobacter crescentus*, scaffold proteins form polarized and dynamic membrane-free signaling hubs via LLPS to facilitate cell fate determination [123]. Thus, LLPS may serve as a general biophysical mechanism regulating cell division of eukaryotic and prokaryotic organisms.

At present, many problems are urgently needed to be solved in the field of biomolecular condensates, and the most critical ones are how to accurately define and investigate phase separation in physiological environment, and how to establish the correlations between phase separation phenomena and biological functions of biomolecules. Although a variety of proteins involved in cell division have been found to exhibit LLPS properties *in vitro*, rigorous studies are required to characterize the biophysical properties and functions of protein condensates *in vivo*. For example, whether SPD-5 undergoes LLPS *in vivo* remains unknown. What are the consequences of disrupting phase separation of PCNT, SPD-5, or PLK-4 during cell division? What’s the signal to initiate assembly and disassembly of protein condensates in extremely precise coordination with the cell cycle? How are the protein condensates recognized and transported by motor proteins along microtubules? What determine the spatiotemporal localization (anchoring mechanism) of protein condensates? More studies are needed before these questions are adequately answered.
